# Bacterial ghosts embedded in natural hydrogels as drug delivery vehicles for cancer treatment

**DOI:** 10.1039/d6ra00738d

**Published:** 2026-02-20

**Authors:** Arzanish Mehmood, Sadia Masood, Talha Farooq Khan, Danial Asghar, Ayeesha Mujeeb

**Affiliations:** a Atta-ur-Rahman School of Applied Biosciences (ASAB), National University of Sciences and Technology (NUST) Islamabad Pakistan arzanishmehmood@yahoo.com; b Department of Materials Science and Engineering, Institute of Space Technology Islamabad Pakistan; c Department of Engineering & Applied Sciences, OntarioTech University Oshawa Canada; d Department of Biological Science and Engineering (BESE), King Abdullah University of Science and Technology, KAUST Saudi Arabia

## Abstract

The growing complexity of cancer treatment requires new drug delivery systems that improve the effectiveness of therapy and reduce adverse effects. This study explores the potential of using bacterial ghosts (BGs) in combination with hydrogels to develop a targeted drug delivery system for cancer treatment. BGs, based on non-pathogenic bacteria, offer several distinct advantages, including biocompatibility, maintenance of immunogenicity, and effective encapsulation of therapeutic agents. The BG-hydrogel system enhances stability and controlled release of encapsulated drugs, which enhances the therapeutic window of drugs. Herein, the BGs were loaded with the anticancer drug doxorubicin (DOX) and subsequently encapsulated in four different hydrogels, namely agarose, agar, aloe vera, and sodium alginate, to produce a Bacterial Ghost-Hydrogel System (BG-HS). The BGs, DOX-loaded BGs, hydrogels, and BG-HS morphologies were characterized by Scanning Electron Microscopy (SEM), revealing distinct structural features conducive to drug delivery applications. Detailed chemical analysis was conducted using Fourier Transform Infrared Spectroscopy (FTIR), confirming the presence of all individual components in the BG-HS. UV-vis spectroscopy demonstrated a pH-responsive drug-release profile attributed to hydrogel ionization in acidic and basic solutions. Compression testing was used to evaluate the mechanical integrity of hydrogels for *in vivo* applications. Also, a macroscopic diffusion experiment with a model solute (Rhodamine-6B) was performed to identify the hydrogel with the highest transport performance. The study's results indicate that BGs with natural hydrogels, particularly agarose, are a promising approach for future cancer therapy and warrant further preclinical and clinical research.

## Abstract 2

Schematic overview of the BG–NH system showing morphological characterization, structural analysis, optical profiling, mechanical evaluation, and Rhodamine 6G diffusion studies to assess structural integrity and controlled-release behavior.

## Graphical abstract

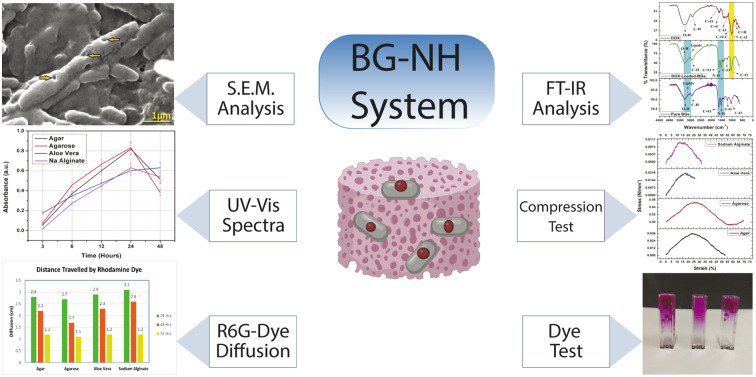

## 1 Introduction

Cancer is a fatal illness and is categorized among the leading causes of death in the world. According to the 2024 report by the World Health Organization (WHO), based on 2022 global statistics, it is estimated that 1 in 9 men and 1 in 12 women die of this disease worldwide.^1^ In 2020, cancer was the second leading cause worldwide, accounting for an estimated 10 million fatalities.^2–4^ Unfortunately, future predictions indicate a significant rise in cancer-related deaths, with estimates suggesting a substantial increase to 17.0 million deaths yearly by 2030.^5^ The high fatality rates of cancer can be attributed, in part, to the limited treatment options available. Primary treatments include chemotherapy, surgery, and radiotherapy, which have been used as standalone or in combination for many decades.^6^ Furthermore, many anticancer drugs have low bioavailability and limited targeting ability, and fail to distinguish between normal and cancerous cells.^7^

More recent developments in cancer treatment include hormonal therapy, immunotherapy, stem cell therapy, and nanotherapeutics. Primarily, nanotherapeutics refers to the use of drug-delivery systems based on nanoparticles to deliver drugs in a controlled, targeted manner.^8^ It harnesses nanomaterials' properties to modify drugs and therapies, thereby altering their biological processes (absorption, cellular uptake, distribution, elimination, and metabolism) and their pharmacological effects.^9^ It is an emerging area of nanotechnology that has the power to completely change the way cancer is treated by using injectable nanoscale drug delivery systems, such as nanoparticles, liposomes, nanotubes, micellar systems, nanospheres,^10,11^ bacterial ghosts (BGs),^12^ and hydrogels.^13^ These drug delivery vehicles (DDVs) provide several advantages over traditional cancer treatments, including localized targeting, enabling drug release at the infected site, outstanding drug-loading capacity,^14^ reduced toxicity to healthy tissues,^15^ enhanced drug solubility, drug stability, and half-life.^16^ Among the various DDVs considered, bacterial ghosts (BGs) were selected for this study due to their potential as advanced drug delivery systems (ADDS). BGs are empty cellular envelopes of Gram-negative bacteria that retain their surface and antigenic properties, including pathogen-associated molecular patterns (PAMPs), which can bind to receptors on cancer cells, allowing BGs to be engulfed by the cancer cells *via* micropinocytosis. As BGs lack the internal components necessary for replication, their large hollow structure allows for easy encapsulation of many therapeutic agents, including peptides, drugs, and DNA, making them polyvalent DDVs with higher loading efficiency than other nanotherapeutics.^17^ Since they are non-living and lack genetic material, they cannot revert to their original forms or transfer pathogenic islands and antibiotic resistance genes.^18^ In contrast, most other DDVs lack inherent immune-activating properties and thus require additional surface modification, such as PEGylation, antibody conjugation, or peptide ligand conjugation, to achieve comparable targeting, making the process considerably costly.

BGs can be produced by chemical and genetic methods. The classical method involves the controlled expression of a phage lysis gene E. However, it is effective only against Gram-negative bacteria; the procedure is technically complicated, expensive, and may still require additional steps to ensure complete inactivation. The chemical ones, including the use of minimal inhibitory concentrations of agents such as NaOH, are less complex but can be applied to both Gram-negative and Gram-positive groups; however, strong alkali may destroy surface antigens and reduce immunogenicity. To minimize this problem, a recent protocol suggested using low concentrations of Tween-80, which lead to a reduction in pH levels caused by lactic acid, to obtain structurally intact BGs that retained their surface properties.^19^

Recent research has highlighted the therapeutic importance of BGs, including their ability to target inflamed tissues and reduce the symptoms of immune-mediated diseases in zebrafish models.^20^ A review by Hajam *et al.* (2017) summarizes numerous *in vivo* experiments in mice, pigs, cattle, poultry, and fish that demonstrate that BGs are non-replicating, non-toxic, low in endotoxicity, and that they also serve as safe vaccines and adjuvant systems. Furthermore, *Salmonella typhimurium* ghosts induced by NaOH have been reported to have decreased cytotoxicity to murine macrophages. They can be safely used in rats following repeated vaccination, which induces protection without causing side effects.

Existing studies confirm that BGs loaded with anticancer drugs effectively target cancer cells and inhibit their growth. Rabea used the Tween-80 method for the first time, then loaded DOX into those BGs for antiproliferative dose testing in HepG2 cells, achieving 64.5% cell mortality.^21^ BG-based DDVs require fewer and less frequent dosages; they are safer and beneficial for the elderly.^18^ However, while BGs offer targeted drug delivery, their dispersion after local administration may reduce drug retention at the tumor site.^22^ To address this issue, hydrogels were incorporated to create a hydrated protective barrier around BGs, preventing their rapid systemic clearance from the body and enabling sustained drug release at the targeted site. Hydrogels can also be used as effective drug loading platforms by controlling their porosity and cross-linking density, as demonstrated in various studies.^23,24^ Furthermore, the drug release rate can be controlled by altering the hydrogel structure in response to environmental stimuli, such as temperature and pH.^25^ Lastly, hydrogels maintain elevated local drug concentrations at the targeted site for an extended period, enhancing therapeutic efficacy.^26^

This study aimed to create a hybrid system by combining drug-loaded BGs with the hydrogels agar, agarose, aloe vera, and sodium alginate for a comprehensive analysis of drug release profiles in 3D cell culture systems. Specifically, these four hydrogels were chosen because they are the most common hydrogel matrices used in biomedical systems, especially drug delivery, owing to their unique gelation mechanisms.^27–29^ Agar and agarose can be used as physically, thermoreversible, cross-linked networks; sodium alginate can be used as an ionically cross-linked network; and aloe vera can be used as a physically cross-linked network. This selection enables a structural comparison of biologically distinct hydrogels with respect to swelling, mechanical stability, and drug-release characteristics under physiological conditions. In addition, these natural hydrogels, in terms of *in vivo* applications, have advantages over synthetic hydrogels, *e.g.* (PEG-based or thermoresponsive); they are generally regarded as biodegradable natural polymer matrices, they can be prepared under mild and non-toxic conditions and do not require any chemical cross-linkers. In contrast, synthetic hydrogels often require chemical modification to incorporate degradable linkages and chemical cross-linkers to achieve compressive strength and controlled *in vivo* biodegradability.^30–32^ However, natural hydrogels have drawbacks, such as reduced mechanical strength, sensitivity to the physiological environment, or compositional variability, which were compared relatively in this study.

The importance of this hybrid system is to overcome two significant shortcomings of traditional chemotherapy: dose-dependent toxicity and non-specific cell killing. In conventional therapy, the doses of the systemic drug required to produce a therapeutic outcome are usually large, which results in non-specific exposure of healthy tissues, leading to dose-related toxicity. The current system involves incorporating potent chemotherapeutic agents into a non-toxic delivery system, thereby enabling localized drug retention and reducing systemic exposure. The hydrogel serves as a local depot that entraps the bacterial ghosts at the tumor site and restricts early drug diffusion. In an acidic tumor microenvironment, the hydrogel network becomes gradually loosened, enabling controlled release of the encapsulated drug. This local and prolonged-release approach minimizes the need for a high systemic dose and enhances tumour-localized drug delivery without the need for additional modifications, unlike other DDVs. A comparative analysis of various natural hydrogels helps identify the most appropriate one for obtaining controlled release and local therapeutic activity.

To the best of our knowledge, this is the first study to investigate the synergistic potential of BGs and hydrogels in a single formulation, combining their respective advantages to optimize drug delivery in tumour therapy. Previous studies have reported effects of incorporating DOX into various hydrogels and nanoparticles, such as peptides^33^ and GelMA;^34^ however, none have examined the impact of incorporating BGs or DOX-loaded BGs into any hydrogel system.

## 2 Materials and methods

The main chemicals and reagents used in the experiment were analytical grade and purchased from various manufacturers. The bacterial strain used to prepare the BGs was sourced from the Microbiology and Technology (MBT) Lab at the Atta-Ur-Rehman School of Biotechnology (ASAB) National University of Science and Technology (NUST). Doxorubicin was purchased from Pfizer, and Tween-80 from AppliChem. All reagents were used without further purification unless otherwise specified.

### 2.1 Production of bacterial ghosts

The primary inoculum was prepared by transferring a single colony of *E. coli* into 25 mL of Luria Broth (LB) and incubating in a shaking incubator for 12–18 hours to promote bacterial growth. Next, a 16% (v/v) Tween-80 lysis medium was made by adding 4 mL of Tween-80 to 21 mL of LB. Subsequently, 1 mL of the overnight culture (*E. coli*) was inoculated into the Tween-80 solution and incubated at 37 °C in a shaking incubator for 24 hours to initiate membrane permeabilization. Afterwards, the pH was adjusted to 3.28 with pre-sterilized lactic acid^35^ to optimize conditions for bacterial lysis. The culture was incubated again at 37 °C for 1 hour, and subsequently, the solution was centrifuged at 4000 rpm for 10 minutes. The supernatant was separated, and the bacterial pellets were washed thrice with half-normal saline (0.45% sodium chloride) to remove residual media and reagents, thereby ensuring purity.^36,37^ The entire experiment was carried out in triplicate.

### 2.2 Determination of DNA content

The NanoDrop 2000/2000c spectrophotometer (Thermo Scientific, MA, USA) was used to determine the quantity of DNA released in the supernatant. The 260 nm/280 nm absorbance ratio was measured to assess DNA purity.^36^ The BGs were lyophilized (freeze-dried) using a bench-top freeze-drier and stored at −20 °C for further use.^12,38^ Fig. S1 provides an overview of the BG preparation process.

### 2.3 Loading of doxorubicin in the bacterial ghosts

To load DOX into the BGs, a 5 mg mL^−1^ DOX solution was prepared, from which 200 µL was used to incubate 10 mg of lyophilized BGs for 10 minutes. Next, 5 mg of DOX was dissolved in 1 mL of Tris–HCl buffer, and the pH was adjusted to 9.0 with 1 M NaOH. The solution was then incubated at 25 °C for 10 minutes. To separate the DOX-loaded BGs, the mixture was centrifuged at 5000 rpm for 3 minutes. The collected pellets were washed three times with the buffer and then freeze-dried for later use.^21,38^ The resulting DOX-loaded BGs weighed a total of 1 mg. Fig. S2 demonstrates the entire process.

### 2.4 Drug release from bacterial ghosts

The release kinetics of DOX from BGs were analyzed under different pH conditions and temperatures to simulate physiological and tumor microenvironments. UV-vis spectrophotometry was used to monitor DOX release at 470 nm. New, non-lyophilized DOX-BG pellets were made, and 3.9 mL of phosphate-buffered saline (PBS) at pH 3.0, 6.5 and 7.4 was added. The PBS solutions were prepared by dissolving PBS tablets in 100 mL of distilled water, then adjusting the pH to 7.4 with 0.5 M HCl (acidic) and 0.5 M NaOH (alkaline). The samples were thoroughly mixed, and 1.3 mL of each was pipetted into each well of a 24-well plate, which was then incubated at 37 °C to replicate physiological conditions.^39^ The release buffer (PBS solution) at the top of each well was sampled at specific intervals (3, 6, 12, 24, and 48 hours) and measured at 470 nm using UV-vis spectrophotometry to determine the amount of DOX released. A concentration-*versus*-absorbance calibration curve was generated to determine the drug concentration at each time point. All measurements were conducted in triplicate and used for creating the cumulative drug release profile using the following steps:

(1) Calculate the release concentration (RC) from the calibration curve at a given time, mcg per mL.

(2) Calculate the amount of drug released, mg = RC mcg per mL × volume (mL)/1000.

(3) Calculate the percentage drug release: % = (amount of drug release/amount of drug loading) × 100.

### 2.5 Preparation of hydrogels

Preparation of the agar hydrogel begins by dissolving 0.3 g of agar in distilled water. The solution was boiled down and left to cool, which formed a solid agar gel.^40^ Likewise, agarose hydrogel was prepared using the procedure reported by^41^ with minor modifications, including dissolving 0.3 g of agarose in 100 mL of distilled water. The solution was then boiled and allowed to cool, forming the agarose gel. Aloe vera hydrogel was purchased from Lark Co. and did not require preparation.

To prepare the 7% sodium alginate hydrogel, 1.0 g of sodium alginate was stirred in 100 mL of distilled water using a magnetic stirrer for 1 hour. The powder was fully dissolved, and the solution pH was adjusted to 6.0 with 1 M HCl. The mixture was autoclaved at 121 °C and 15 psi for 2 hours, to maintain sterility and avoid contamination. Simultaneously, a 0.1 M CaCl_2_ solution was prepared by dissolving 0.1 g of CaCl_2_ in 500 mL of distilled water and ensuring complete dissolution. This solution was autoclaved and stored in the refrigerator at 4 °C. Subsequently, 8.0 mL of the CaCl_2_ solution was mixed with 1.0 g of the sodium alginate solution and vigorously mixed using a vortex machine to form the sodium alginate hydrogel.^42^

### 2.6 Synthesis of BG-HS

After preparing all the natural hydrogels, the next step was to synthesize the BG-HS system. Using a sterile spatula, 3 mL of the as-prepared DOX-loaded-BGs (Section 2.2) were mixed with each of the prepared hydrogels in a falcon tube, ensuring homogenous dispersion of BGs within the hydrogel matrix. Since excessive heat can distort the properties of DOX-loaded BGs, the formulations were dispersed whilst the hydrogels were slightly warm, thereby avoiding any compromise to the structural integrity of the BGs. For sodium alginate, the DOX-loaded-BGs were first dissolved in a calcium chloride solution to facilitate cross-linking. Once fully incorporated, the mixture was blended with the sodium alginate solution, and CaCl_2_ was added to achieve optimal dispersion. After obtaining a homogeneous mixture, the hydrogel samples were transferred into a 24-well culture plate using a pipette.

### 2.7 Drug release from BG-HS

A DOX release study was conducted to evaluate the pH-dependent drug release profile of BG-HS. To assess the release profiles under different conditions, two pH levels were chosen: pH ∼6.5 simulating a tumor microenvironment and pH ∼7.4 representing normal physiological conditions. Buffer solutions were prepared by dissolving PBS tablets in distilled water, and pH was adjusted with 0.5 M HCl for acidic conditions (pH 6.5) and 0.5 M NaOH for alkaline conditions (pH 7.4). 500 µL of BG-HS were placed in a 24-well plate. Once the gels were set, 1.3 mL of PBS (pH 6.5 or 7.4) was poured over the gels, and the plates were incubated at 37 °C for 3, 6, 24, and 48 hours to simulate *in vivo* conditions. Drug release was monitored at these set time intervals using UV-vis spectroscopy at 485 nm, with triplicate measurements ensuring accuracy.^39^ The cumulative drug-release curve was generated using eqn S1, enabling quantification of DOX in the collected buffer. This process is visually represented in Fig. S3.

### 2.8 Characterization of BGs, DOX-loaded-BGs, plain hydrogels and BG-HS

#### 2.8.1 Scanning electron microscopy analysis (SEM)

The morphologies of the as-prepared lyophilized BGs, DOX-loaded BGs, plain hydrogels, and BG--HS were examined using the SEM (JSM5910 JEOL, Japan). All samples of BGs and hydrogels were prepared in a dried/lyophilized state for SEM analysis, which was conducted repeatedly after synthesis of the BG-HS components.

#### 2.8.2 Fourier transform infrared spectroscopy analysis (FT-IR)

FT-IR spectroscopy was used to examine the chemical composition of BGs, hydrogels, DOX, DOX-loaded-BGs, and BG-HS. Spectra were collected in the 400–4000 cm^−1^ wavelength range using a Thermo Scientific Nicolet 6700 FT-IR spectrometer equipped with a diamond crystal and set to attenuated total reflectance (ATR) sampling.

#### 2.8.3 Compression testing

The mechanical properties of the hydrogels were measured using a Galdabini Quasar 50 universal testing machine, equipped with a 100-N load cell (AG-X plus, Italy). A dynamic cyclic compression test was performed on the samples within a strain range of 0–70% and a compression rate of 1.0 mm min^−1^, continuing until fracture. The samples were prepared as cylinders with diameters and thicknesses of 10 mm. 3.5 mL of each gel was uniformly moulded in a 10 mL syringe with a 10 mm diameter. The resulting stress–strain curve was derived, and measurements were averaged to determine the outcomes.^43^

#### 2.8.4 Macroscopic diffusion experiments

Diffusion tests were conducted on the hydrogel samples to study the transport mechanism of Rhodamine 6-G dye within the hydrogels. The polymethyl methacrylate (PMMA) cuvettes were filled with the hydrogel and submerged in a solution containing Rhodamine 6B dye as a model solute. UV-vis absorption spectra were obtained at 24, 48, and 72 hours to study the diffusion process. The distance travelled by the dye was measured from the base of the cuvette to the point where diffusion stopped. The mean values and standard deviations of these parameters were calculated by averaging the results across three diffusion times.^43^

## 3 Results and discussion

### 3.1 Characterization and confirmation of BGs

O.D. measurements indicated that the *E. coli* culture with an O.D. above 0.4 exhibited poor lysis upon addition of Tween-80. In contrast, *E. coli* cultures with an O.D. of 0.2–0.3 produced highly effective bacterial ghosts.

### 3.2 Quantification of DNA in BGs

The remaining DNA in the BG pellets was measured at 1500–1700 ng µL^−1^, compared with the untreated bacteria, which had a concentration of 400–700 ng µL^−1^. Additionally, the mean concentration of genomic DNA in the BG supernatant was 1587 ng µL^−1^.

### 3.3 Viability assessment of BGs

After 72 hours of incubation, the untreated *E. coli* cells showed abundant growth on nutrient agar plates. In contrast, the Tween-80-treated cultures showed no viable bacterial cells after incubation.

### 3.4 Scanning electron microscopy (SEM)

#### 3.4.1 Detection of *E. coli* DH5α formation by SEM

**Fig. 1:**
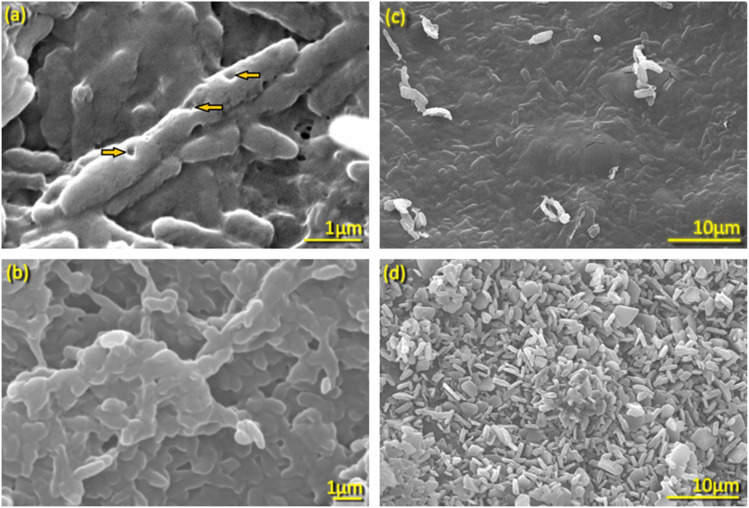
Scanning electron micrographs of (a) and (c) the as-prepared pure bacterial ghosts, and (b) and (d) DOX-loaded bacterial ghosts. The arrows indicate porosity on the surface of as-prepared BGs.

The structural morphology of the prepared BGs was analyzed using SEM, which corroborated characteristics consistent with those reported in previous studies.^36,37^ The *E. coli* ghosts had small surface pores with an average diameter of 100–140 nm, as shown by the arrows in Fig. 1(a and c). These pores are channels through which cellular components, such as cytoplasmic material and DNA, can exit. As a result of this removal, the *E. coli* ghosts appeared more translucent, flattened, and elongated. The depicted BGs measured approximately 3–4 µm in length and 1 µm in width. Transmembrane tunnels were observed on the surface of BG cells capable of facilitating the extrusion of the DNA and protein contents.^36^

#### 3.4.2 SEM of unloaded and loaded hydrogels

**Fig. 2:**
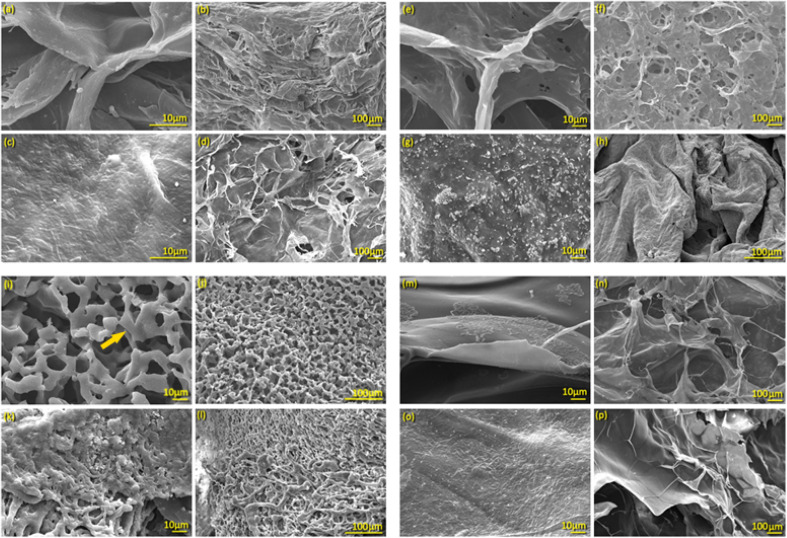
SEM of (a) and (b) plain agar; (c) and (d) DOX-BGs loaded agar; (e) and (f) plain agarose; (g) and (h) DOX-BGs loaded agarose; (i) and (j) plain aloe vera; (k) and (l) DOX-BGs loaded aloe vera; (m) and (n) plain sodium alginate; (o) and (p) DOX-BGs loaded sodium alginate.

Fig. 2 depicts the structure and morphology of the four plain hydrogels and the hydrogels loaded with BGs and DOX. The typical pentagonal structure of the plain agar hydrogel is clearly visible in Fig. 2(a and c), and the overall morphology matches previously published data.^40^ On the other hand, the DOX-BGs-agar sample showed additional layers in the same morphology, as shown in Fig. 2(b and d). In contrast to agar alone, the DOX-BG loaded hydrogel had a more interconnected cell network, retaining distinct characteristics, such as a uniform dispersion of bacteria, resulting in a homogeneous appearance.^44^ SEM images confirmed that bacterial cells were embedded within the hydrogel matrix rather than freely dispersed.^44^ The texture of the hydrogel matrix affected the spatial arrangement of bacteria, with cells occupying spaces and adhering to the matrix. The clarity of the hydrogel, coupled with the visible presence of bacteria, provided insights into bacterial interactions with the gel matrix.^44^

Agarose hydrogel, as shown in Fig. 2(e and g), exhibited expansive layers/sheets and contained large pores, as reported by Jayawardena *et al.* (2023). After loading DOX, the pore size decreased due to bacterial integration in the gel matrix.^45^ The hydrogel surface became rough, suggesting a strong interaction between the *E. coli* and agarose gel.^46^

The morphology of the aloe vera hydrogel displayed characteristic fiber-like microstructures embedded within the hydrogel and aligned parallel along the surface plane, as illustrated in Fig. 2(i and k). SEM images of loaded aloe vera in Fig. 2(j and l) exhibited plate-like morphologies on the surface as indicated by the arrow in Fig. 2(j). The roughness of the film surface is attributed to bacterial aggregates dispersed on it.^47^

SEM images of the sodium alginate composite hydrogel showed a densely compacted, rough surface structure with pores measuring approximately 50 µm. It exhibited a uniform microstructure with interconnected, well-defined porosity and high pore density,^48^ as seen in Fig. 2(m and o). Once loaded, bacterial rods were fully embedded in the gel network, as observed at 10 µm and 100 µm Fig. 2(n and p).

### 3.5 FTIR analysis

#### 3.5.1 Doxorubicin

**Fig. 3:**
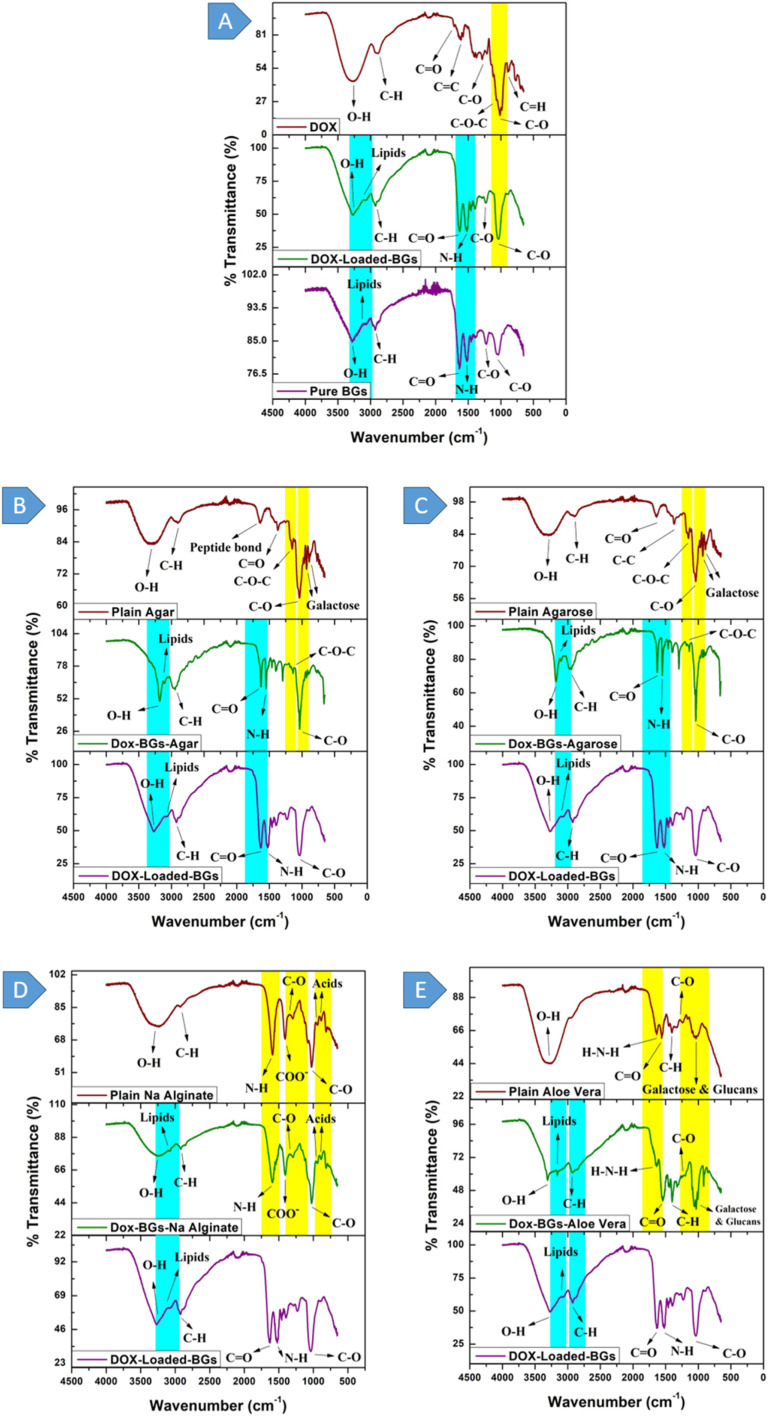
FTIR for unloaded (plain) and DOX-loaded samples: (A) bacterial ghosts, (B) agar, (C) agarose, (D) sodium alginate, and (E) aloe vera.

The FTIR spectroscopy of pure DOX, shown in Fig. 3(a), displays distinct peaks that indicate specific molecular vibrations. O–H stretching vibrations are evident at 3320–3230 cm^−1^,^49^ which are linked to hydroxyl groups. Methoxyl groups are identified by a peak around 2897 cm^−1^.^50^ The peaks of 1700 cm^−1^ are related to C=O stretching, and 1616 cm^−1^ is related to (C=C) stretching.^50^ Also, the C–O bonding is indicated by the 1288 cm^−1^ peak, whereas the shoulder indicates C–O–C bonding at 1074 cm^−1^.^50^ Lastly, C–O is linked to the bond at 1026 cm^−1^.^51^

#### 3.5.2 Bacterial ghosts

FTIR validated the synthesis process of BGs and hydrogels. Additionally, it confirmed the presence of molecular groups in DOX-loaded BGs and their incorporation in hydrogels through characteristic absorption bands, as shown in Fig. 3(a). A broad band peaking at 3300 cm^−1^ corresponds to O–H stretching vibrations.^52^ The region between 3000–2800 cm^−1^ is primarily characterized by the C–H stretching vibrations found in fatty acids.^52^ At 2930 cm^−1^, fatty acids of various membrane components were demonstrated with a functional group of (C–H).^53^ This reflects the characteristic C–H stretching vibration of the aliphatic carbon chain, highlighting a fundamental aspect of the *E. coli* cell wall, which includes fatty acids, membrane proteins, and other amphiphilic molecules.^54^ The absorption bands at 1643 cm^−1^ and 1544 cm^−1^ correspond to the amide I and II bands present in various proteins and peptides.^52^ The bands visible at 1200–900 cm^−1^ correspond to the RNA/DNA region, with vibrations arising from PO^2−^.^52^ The intense vibration at 1049 cm^−1^ corresponds to the asymmetric stretching mode of P=O, which is associated with a combination of phospholipids and esters.^55^

#### 3.5.3 Doxorubicin loaded with bacterial ghosts

The FTIR spectrum shows prominent peaks related to the loading of DOX, as shown in Fig. 3(a). The yellow bar represents a specific C–O group present only in DOX. The two blue bars indicate unique groups found in BGs, which include OH–, lipids, C=O, and N–H. Upon addition of DOX to BGs, the yellow and blue bars, which represent distinct functional groups, were observed to co-exist, which is a confirmation that DOX has been loaded into BGs.

#### 3.5.4 DOX-BGs-agar

Fig. 3(b) shows the FTIR spectrum of plain agar, which has some characteristic absorption bands that give information on the chemical composition of the agar. Specifically, the O–H stretching^56^ is represented by the high absorption peak at 3323 cm^−1^, which indicates that the number of hydroxyl groups in agar is high.^57^ The peak at 2900 cm^−1^ evidences the presence of the methoxyl groups.

The band near 1643 cm^−1^ is associated with the stretching vibration of the conjugated peptide bond formed by the amine (NH) and acetone (CO) groups. Additionally, the peak at 1370 cm^−1^ is attributed to ester sulfate.^58^ The intense absorption region centred at 1030 cm^−1^ can be attributed to the stretching vibrations of the C–O pyranose ring, which is characteristic of all polysaccharides.^59^ Another notable feature is the C–O–C stretch, observed at 1153–1156 cm^−1^, indicating the presence of carbohydrates.^60^ The peak at 928 cm^−1^ can be attributed to the C–O vibration of the 3,6-anhydrogalactose residue.^59^ At the same time, the bands at 887 cm^−1^ correspond to the C–H bending at the anomeric carbon in β galactopyranosyl residue.^59^ In the FTIR spectrum, the two distinctive peaks or chemical bonds of plain agar gel are symbolized by the yellow bars, and the bonds unique to DOX-loaded BGs are represented by the blue bars. Once the loading process is complete, both the yellow and blue bars are visible in the spectrum when DOX-loaded BGs are encapsulated within the agar hydrogel.

#### 3.5.5 DOX-BGs-agarose

The FTIR Spectrum of plain agarose, Fig. 3(c), shows clear absorption bands, which give us an insight into the chemical composition and structure of agarose. The O–H stretching vibrations are connected with the absorption band around 3400 cm^−1^.^61^ The sharp peak at 3400 cm^−1^ indicates the presence of hydroxyl groups. The signal at 2943 cm^−1^ is linked with the sp^3^ C–H stretching vibrations, which are characteristic of aliphatic carbon chains.^61^ C–H bonds are characteristic of different organic compounds. At 1370 cm^−1^, a distinct band is observed, corresponding to C–C bending.^61^ This bending mode is related to the structural arrangement of carbon atoms in the agarose molecule. The absorption band at 1643 cm^−1^ can be attributed to the C=O stretching vibration of carbonyl groups (C=O) present in the sugar units of the agarose molecule and its saccharide structure.^62^ The presence of carbonyl groups is an important structural characteristic of agarose.

Additional notable features in the spectrum include the IR bands at 1158 cm^−1^ and 1071 cm^−1^, corresponding to –C–O–C– and glycosidic linkage (C–O), respectively. These bands provide details on the glycosidic bonds of the agarose structure, a polysaccharide.^63^ The absorption bands are assigned to 3,6-anhydrogalactose at 932 cm^−1^ and 890 cm^−1^.^63^ These bands are typical of this sugar unit and provide more information about the composition of agarose. The FTIR spectrum highlights specific features that distinguish agarose hydrogel from DOX-loaded BGs. The two yellow bars represent the region shared by agarose hydrogel, while the two unique blue bars found in DOX-loaded BGs are derived from the BGs. These unique peaks in DOX-loaded-BGs likely correspond to specific functional groups or chemical components associated with the bacterial ghost structure.

#### 3.5.6 DOX-BGs-aloe vera

The FTIR spectra of aloe vera hydrogel, shown in Fig. 3(e), revealed peaks within the range of 3283–3466 cm^−1^. These peaks are associated with the stretching vibrations of hydroxyl groups and confirm their presence. The peak at 1637 cm^−1^ is attributed to the presence of the amino groups in alcohols, phenols and amines in the aloe vera extract. Also, the band at 1402 cm^−1^ is associated with the C–H stretching of alkanes,^64^ indicating the presence of aliphatic carbon chains. The carbonyl group^65^ is a significant structural element and is represented by the peak at 1558 cm^−1^. Additionally, the absorption band at ∼1236 cm^−1^ is characteristic of the C–O stretch of carboxylic acids.^66^ Significantly strong bands within the range of 1076–1034 cm^−1^ strongly indicate the presence of polysaccharide sugars, such as galactose and glucans.^65,67^ In the spectral analysis, two distinct features, marked by yellow bars, are shared between aloe vera hydrogel and DOX-loaded-BGs. Furthermore, two unique features, indicated by blue bars, are specific to DOX-loaded-BGs. The presence of these blue peaks is directly attributed to the inclusion of BGs within the sample. Notably, these distinct blue peaks become visible only when DOX is loaded into the BGs.

#### 3.5.7 DOX-BGs-Na alginate

The FTIR spectra investigation of sodium alginate, illustrated in Fig. 3(d), contains a stretching vibration of the OH group at 3600–3000 cm^−1^ and –CH vibration bands at 2930–2845 cm^−1^. The symmetric and asymmetric stretching vibrations of the COO– groups were attributed to the bands seen at ∼1422 cm^−1^.^68^ The band observed at 1598 cm^−1^ is attributed to a N–H bending vibration.^69^ The Ca^2+^-induced cross-linking of sodium alginate altered the COO^−^ stretching bands, which resulted in a reduction in the intensity and wavenumber of the carbonyl peak. This reduction indicates cross-linking and is further supported by evidence of a shoulder at 1080 cm^−1^ and lengthening of C–C and C–O bonds.^68^ The peak at 1296 cm^−1^ corresponds to C–O.^70^ The bands around 1030 cm^−1^ (C–O–C stretching) are attributed to sodium alginate's saccharide structure.^71^ The stretching vibration bands observed at 816, 887, and 945 cm^−1^ are typical of mannuronic and guluronic acids.^68^ Additionally, slight alterations in carboxyl groups revealed ionic interactions between Ca^2^ and sodium alginate,^68^ underscoring the chemical changes during cross-linking. The blue bar underscores the distinct bonds found within DOX-loaded-BGs, while the three yellow bars emphasize the unique bonds within the sodium alginate hydrogel. Once DOX-loaded-BGs are integrated into the sodium alginate hydrogel, all four bars, consisting of one blue and three yellow, become collectively visible.

### 3.6 *In vitro* release assay of doxorubicin from bacterial ghosts

**Fig. 4:**
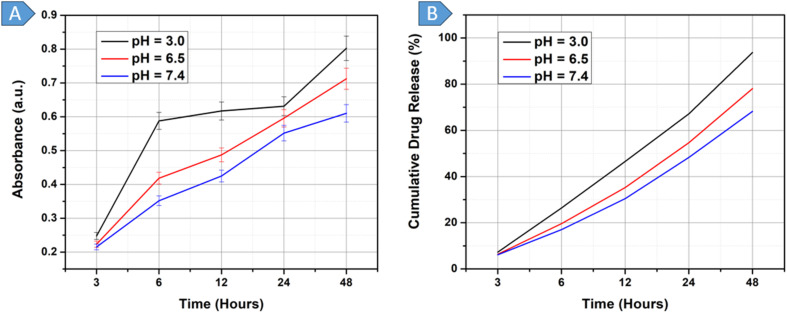
(A): *In vitro* release of drug from BGs at pH 3.0, 6.5 and 7.4, (B): cumulative drug release % from BGs at pH 3.0, 6.5 and 7.4.

The DOX-loaded-BGs underwent an *in vitro* drug release assay at pH 3.0, 6.5, and 7.4 over 48 hours based on the time intervals: 3, 6, 12, 24, and 48 hours, as shown in Fig. 4(a), using an absorbance *vs.* time graph. The cumulative percentage release of the drug was calculated using eqn (1), which uses absorbance values, as depicted in Fig. 4(b).1
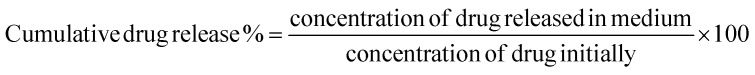


At pH 3.0, the drug-release kinetics exhibited distinct patterns over time. The first stage of the graph shows a burst release from 3 to 6 h, with a total of 26.37% DOX released from the BGs. This rapid, pronounced release is attributed to the environment's extremely acidic conditions, which destabilize the outer members of the BGs. The second stage of the graph, from 6 to 24 h, represents continuous drug release, resulting in an additional 40.77% release, bringing the cumulative release to 67.14%. This phase is attributed to the moderate membrane permeability of BGs, enabling easier diffusion of molecules such as the drug. Finally, between 24 and 48 h, the drug release rate increased once more due to the complete rupture of the BGs, leading to the release of a substantial amount of the remaining drug and yielding a cumulative release of 93.73%, as depicted in Fig. 4(b). This pattern was similarly evident in a study of gelatin-based nanoparticles, which also evaluated drug release at pH 3.0.^72^

Next, the drug release profile of DOX-loaded BGs was observed at pH 6.5 to simulate the mild acid microenvironment of tumors. The initial trend of the absorbance *vs.* time graph in Fig. 4(a) was similar to that obtained at pH 3.0, indicating a burst release between 3 and 6 h. In this period, a total of 19.61% of the drug was released, as shown in the corresponding cumulative percentage *vs.* time graph in Fig. 4(b). The second, consistent phase of the graph from 6 to 48 hour shows an almost constant release rate, resulting in a cumulative DOX release of 78.06%. In acidic solutions (pH 6.5), the carboxylic group predominantly exists in its unionized form, –COOH. It is present in this form without any net charge and does not cause any electrostatic repulsion in the hydrogel. Consequently, the hydrogel holds less water and swells less, resulting in a smaller network structure and pores. The absence of electrostatic repulsion and a tighter hydrogel network effectively capture and retain the drug molecules, resulting in a controlled and gradual release of the drug.^73^ The trend of gradual, nearly constant drug release from the hydrogels is consistent across both graphs in Fig. 4(a) and (b). A similar study investigated the effect of pH 6.5 on DOX-loaded BGs in *E. coli*, suggesting that at this pH, elevated levels of cyclopropane fatty acids in the bacterial membrane increase membrane rigidity, thereby protecting cells from low pH stress. This increased rigidity reduces membrane permeability, limiting the uptake of DOX into BGs. Furthermore, since DOX is ionized at an acidic pH and remains unionized at alkaline pH, its transport across the membrane is more efficient in its unionized form.^74^

The third DOX-loaded BG sample was subjected to a drug-release assay at pH 7.0, which mimics the conditions found in blood or normal tissue. The drug release profile can be broken into three stages. Initially, there was an instantaneous burst of drug release from 3 to 6 h, during which 17.01% of the encapsulated drug was released from the BGs. This is followed by a sustained release phase from 6 to 24 h, resulting in an additional 31.29% of drug release, bringing the total drug release to 48.30%. From 24 to 48 h, there was a noticeable decrease in the rate of release. This decrease is due to the significant portion of the drug already being released during the first two phases. A similar study investigating the drug release profile of DOX from BGs at pH 7.4 found that 50% of the drug was released within the first 12 h.^21^ This rapid release is attributed to DOX being in its unionized form at higher pH (alkaline conditions, including pH 7.4), which allows it to cross membranes more easily. Additionally, the reduced electrostatic attraction between DOX and the BG membrane at this pH further facilitates faster release than at lower pH.^74^

Analyzing the drug release trends obtained across all three pH values, it can be extrapolated that the BGs at pH 3.0 exhibited excessive burst release in the initial hours of the experiment. In just the initial 6 h, 26.37% of DOX was released. This is not suitable for drug administration inside the human body for tumor treatment. Therefore, pH 3.0, which is highly acidic, was not to be included in the future drug-release tests of the hydrogels. Instead, the scope was adjusted to focus on pH 6.5 and 7.4. A controlled and sustained release of the drug was observed at pH 6.5, which is similar to the conditions found in cancerous tissue. This suggests that the aforementioned hydrogels can effectively deliver drugs to cancerous cells in a controlled manner. On the other hand, at pH 7.0, which represents the condition of normal tissue, there was an initial rapid release of the drug, followed by a reduction in the rate of drug release. This behavior could have implications for delivering drugs to cancerous tissue; less stringent selection could potentially lead to reduced effectiveness in treating targeted cancer cells and an increased risk of impacting healthy tissue.

### 3.7 *In vitro* drug release profile of BG-HS at pH 6.5

**Fig. 5:**
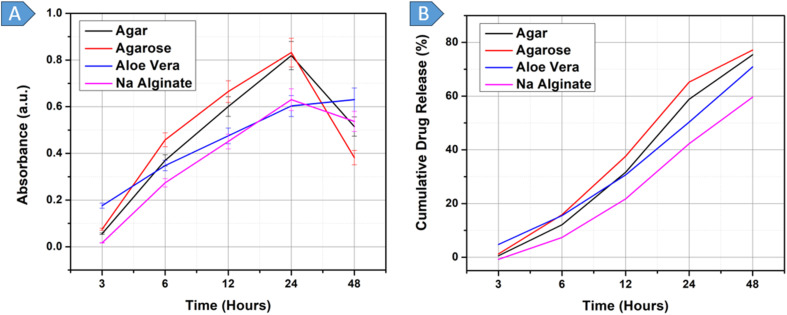
(A): UV-vis spectra showing the release of drug from bacterial ghosts encapsulated in different hydrogels at pH 6.5, (B): cumulative drug release (%) from bacterial ghosts encapsulated in different hydrogels at pH 6.5.

Fig. 5(a) represents the UV-vis spectra displaying the release of DOX from all four hydrogels in the pH 6.5 solution. In contrast, the graph in Fig. 5(b) depicts cumulative percentage of drug release over time for this solution. All the hydrogels exhibited similar patterns during DOX release, with three distinct phases. Phase one shows an initial burst of DOX release with a large percentage of drug released per hour, usually until the 6-hour mark. This phase occurs because of the desorption and diffusion of the drug from the DOX-loaded hydrogel surface, driven by the steep drug concentration gradient between the release medium and the hydrogel surface. The second phase exhibited controlled drug release until the 24-hour mark. As the incubation time increased from 6 to 24 h, the drug-loaded hydrogels underwent progressive swelling, and the drug was released at a slow, constant rate, driven by the concentration gradient established between the hydrogels and the release medium (PBS). The third phase commences after 24 h and is characterized by a reduction in the rate of drug release. This reduction is due to most of the drug having already been released during the preceding phases, leaving only a residual amount within the hydrogel matrix, which is gradually released until the 48-hour mark.

Fig. 5(a) and (b) show that the agar hydrogel had the second-highest drug release rate, like that of agarose. The DOX-BGs-agar formulation exhibited a biphasic release profile, with a first burst phase between 3 and 6 h, during which it released 12.09% of DOX. It was then followed by a controlled, sustained-release period between 6 and 24 h, during which an additional 48.74% of DOX was released gradually, resulting in a cumulative release of 58.83%. The release rate decreased significantly after 24 hours, resulting in a total release of 75.41% over 48 hours. The decrease in release rate over time is consistent with first-order kinetics, implying that drug release is related to the remaining drug concentration in the hydrogel matrix.

Agar hydrogel is highly porous, allowing rapid water absorption and leading to an initial burst release in the early stages of drug delivery. It exhibits pH-responsive swelling behavior due to the ionization of carboxylate groups in the polymer matrix. According to previous studies, swelling intensifies with increasing pH, potentially altering drug-release rates. However, agar hydrogels retain their high water-holding capacity, thereby enabling prolonged drug release. Agar, being an anionic hydrogel, has low interaction with neutral or similarly charged drug molecules. Therefore, diffusion is the primary mode of drug release, leading to a controlled release phase following the initial burst. Lastly, agar hydrogel can hydrolytically degrade, which leads to progressive loss of mass as time goes by, which can further facilitate prolonged drug delivery.

Agarose was the highest-DOX-release gel among the tested hydrogels, exhibiting a biphasic release profile comprising an initial burst release phase followed by sustained diffusion-controlled release. The drug release profile of DOX-BGs-agarose hydrogel was minimal during the initial 3 h, then a major release occurred between 3 and 6 h, resulting in a cumulative release of 15.82%. This rapid release has been attributed to the large pore size of agarose hydrogels, which enhances water uptake and the diffusion of surface-bound drug molecules. This was followed by a decreased release rate, with a controlled release pattern of 3.63 per hour between 6 and 12 hours, then 2.30 per hour until the 24-hour mark. The last period (24–48 hours) had a much lower release rate (0.50%/h), with a cumulative release of 77.19% at the end of the 48-hour study. The Higuchi model (*r*^2^ = 0.9887) best explained the release kinetics, indicating that diffusion through the porous matrix was the primary mechanism controlling drug release.

Agarose hydrogel has large pore sizes, allowing rapid water uptake and causing an initial burst release during the initial stages of drug release. This burst release is mainly observed in the initial hours, as the hydrogel rehydrates rapidly. Thereafter, drug release transitions to a diffusion-controlled process, maintaining a relatively uniform rate over the subsequent hours (6–12 hours). The swelling behavior of agarose remains largely pH-independent, ensuring consistent expansion under varying pH environments. Due to its neutral nature and lack of ionizable functional groups, agarose hydrogels exhibit minimal electrostatic interactions with the drug. The absence of ionic bonding means that drug release is predominantly governed by diffusion rather than chemical interactions, leading to a prolonged, controlled release phase following the initial burst.^75^

The drug release profile of DOX-BGs-aloe vera hydrogel exhibited a distinct biphasic pattern, characterized by an initial burst release phase (0–6 h), a sustained release phase (6–24 h), and a slower terminal release phase (24–48 h). Within the first 6 h, 15.51% of DOX was released from the aloe vera hydrogel, enabling rapid water absorption and immediate drug diffusion. The release rate then gradually decreased, with an additional 34.82% of DOX released between 6 and 24 hours, totalling a cumulative release of 50.33%. The release rate slowed further in the last stage, adding 20.58% to a cumulative drug release of 70.91%. This slowing of the release rate over time is a sign of a diffusion-controlled process, as Higuchi release kinetics would predict, rather than an erosion- or swelling-controlled process.

Within the first 6 hours, aloe vera hydrogel exhibited rapid DOX burst release. This is primarily due to its highly porous nature, loose gel matrix, and lack of crosslinking, which enable immediate drug diffusion from the hydrogel into the surrounding medium, resulting in a burst effect. As it consists mainly of neutral polysaccharides (*e.g.*, acemannan, glucomannan, *etc.*), which do not have significant protonation or deprotonation sites that would respond to pH changes, aloe vera hydrogel did not display substantial pH-responsive behavior. Following the initial burst, the drug release rate progressively declined, transitioning into a sustained release phase. During this period, passive diffusion predominated as the mechanism of release, supported by partial degradation of the hydrogel matrix while maintaining residual structural integrity. The absence of strong ionic interactions and cross-linked polymer chains allowed for continued drug diffusion, albeit at a reduced rate compared to the burst phase. In the third stage, the rate of drug release declined further due to the progressive degradation of the aloe vera matrix, resulting in a weakened matrix structure and larger drug reservoirs that prolonged diffusion times. Moreover, as the swelling and hydration of the aloe vera hydrogel reached saturation, the diminishing concentration gradient reduced the driving force for diffusion, further contributing to the observed deceleration in drug release.^76^

The DOX-BGs-sodium alginate hydrogel exhibited a similar biphasic release profile, consisting of an initial slow burst phase (3–6 hours), a sustained diffusion-controlled phase (6–24 hours), and a final reduced release phase (24–48 hours). Among the four hydrogels assessed, the sodium alginate hydrogel demonstrated the lowest drug release, with only 7.38% of DOX released during the initial burst—the least among all tested formulations. The sustained phase contributed an additional 34.93% release between 6 and 24 h, bringing the cumulative release to 42.31%. In the final phase (24–48 hours), the release rate declined further, reaching a total cumulative release of 59.65% by the end of the 48-hour study period. This drug release profile also closely aligns with the Higuchi diffusion model.

Sodium alginate hydrogel has a highly porous structure, which facilitates rapid absorption and contributes to the initial burst release of the drug within the first three hours. This accelerated release is attributed to the steep diffusion gradient between the hydrogel and its surrounding medium, promoting the rapid desorption of surface-localized drug molecules.^77^ In addition to the burst effect, the swelling pattern of sodium alginate hydrogels is pH-dependent and is mainly determined by the ionization of carboxylate (–COO–) groups. During the experiment, a pH 6.5 buffer solution was added to the hydrogel to ensure a controlled ionization environment. At this pH, slightly above the pKa of sodium alginate (3.555), moderate ionization occurs, leading to electrostatic repulsion between the polymer network's charged groups. This repulsion prevents hydrogel shrinkage while promoting controlled swelling, thereby stabilizing pore dimensions and enabling a sustained, gradual drug release.^78^ Furthermore, sodium alginate forms ionic crosslinks with divalent cations such as Ca^2+^, thereby stabilizing its mechanical properties and modulating drug diffusion. These electrostatic interactions between the hydrogel matrix and charged drug molecules act as a diffusion barrier, slowing drug diffusion and prolonging the controlled-release phase after the initial burst.^79^

The drug release rate is governed by the intrinsic physical and chemical characteristics of hydrogels, including porosity, swelling behavior, and drug–gel molecular interactions.^80^ Porosity dictates the diffusional pathways within the hydrogel matrix, whereby higher porosity facilitates accelerated drug release. Swelling behavior affects drug mobility through controlling water uptake, which may enhance or restrict diffusion based on the hydrogel's ionic interactions and polymer network. Moreover, molecular forces between the drug and the hydrogel matrix, including electrostatic, hydrogen-bonding, and hydrophobic interactions, are essential in controlling drug retention and release kinetics, which ultimately determine the overall release profile.

### 3.8 *In vitro* drug release profile of BG-HS at pH 7.4

**Fig. 6:**
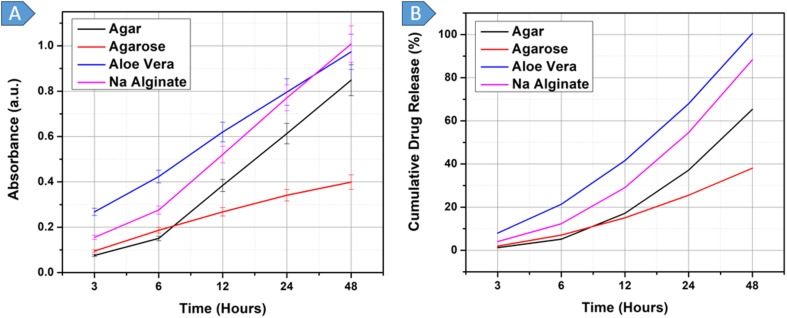
(A): UV-vis spectra showing the release of drug from bacterial ghosts encapsulated in different hydrogels at pH 7.4, (B): cumulative drug release (%) from bacterial ghosts encapsulated in different hydrogels at pH 7.4.

This section analyzes the drug-release profile of DOX from a hydrogel-based hybrid system in a pH 7.4 solution, which represents normal blood pH. The drug release pattern was influenced by similar chemical and physical factors as described in section 3.6. Fig. 6(a) depicts the absorbance *vs.* time graph for DOX-loaded-BGs in different hydrogels, while Fig. 6(b) displays cumulative drug release percentage *vs.* time. An initial minor increase in drug release precedes a prolonged period of continuous drug release up to either 24 or 48 hours. DOX-loaded hydrogels exhibit higher release rates at pH 7.4 when compared to pH 6.5, primarily due to enhanced swelling and expanded pore structure at the higher pH. Under mildly acidic conditions (pH 6.5), hydrogel swelling was limited because ionization is pH-dependent. In contrast, at an alkaline pH (pH 7.4), increased ionization led to greater electrostatic repulsion between carboxylate groups and expanded gel network (larger pore size), thereby increasing the swelling and the rate of drug release.^81^

Agar exhibited an initial drug release of 5.13% up to the 6-hour mark, as shown in Fig. 6(b). Following this, the release rate progressively increased over time, continuing through to 48 hours. Between 6 and 12 h, an additional 12.02% of the drug was released, followed by 20.0% between 12 and 24 h, and 28.19% between 24 and 48 h. This resulted in a total cumulative drug release of 65.34% over the 48 hours.

DOX-BGs-agar hydrogel demonstrated a highly controlled, sustained drug release profile, driven by a gradual diffusion mechanism within its dense yet porous polymeric matrix. The closely knit polymer chains initially limit drug mobility, reducing immediate burst release, even though the hydrogel can absorb water. When swelling occurs, water diffusion increases the size of the polymer network, thereby progressively enlarging pore size and enabling the formation of effective diffusion channels. Unlike hydrogels, whose structure is loosely organized and thus can easily swell and release their contents without control, agar hydrogels exhibit a regulated swelling rate, maintaining a constant diffusion process over the long term. The pH-responsive character of agar also contributes to this mechanism. At pH 7.4, ionization of agar chains induces electrostatic repulsion, thereby loosening the polymer network and facilitating increased drug mobility. This gradual structural relaxation enables a diffusion-driven release mechanism in which concentration gradients act as the primary driving force for drug movement.^82^

DOX-BGs-agarose hydrogel exhibited an almost constant drug release rate from 3 to 48 hours, with notably lower release percentages at each interval compared to those of other hydrogels. At pH (7.4), agarose exhibited a controlled release profile, effectively preventing a sudden release of drugs into the systemic circulation. Fig. 6(b) shows that the drug release was 5.88% at 3–6 h, 7.96% at 6–12 h, 10.51% at 12–24 h and 12.53% at 24–48 h with a total cumulative release of 38.07%. These results indicate the ability of agarose to provide a stable and continuous supply of drugs for an extended period of time. The percentage of drug release at every time interval was significantly lower with agarose than with the rest of the hydrogels, which underscored the ability of agarose to release drugs in a controlled and gradual manner. This controlled drug release characteristic positions agarose as a promising candidate for formulations where excessive or premature drug release must be avoided.

DOX-BGs-agarose exhibited a highly controlled and sustained drug-release profile, driven by a gradual diffusion process superior to that of the other hydrogels tested. Its dense yet porous polymeric network restricted drug mobility, resulting in a slow, steady release phase. As the hydrogel absorbed water, swelling gradually increased the pore size, creating more diffusion pathways for drug release. The non-ionic and neutral agarose nature allowed it to remain stable in its swelling behavior and not to be affected by pH-dependent ionic interactions. Agarose is highly hydrophilic and therefore retains water within its structure, creating a hydrated network that acts as a diffusion barrier and prevents an immediate, uncontrolled burst of the drugs. Unlike other hydrogels that undergo rapid swelling and sudden drug release, agarose exhibited controlled swelling, supporting a consistent, gradual diffusion process. The absence of strong chemical interactions between DOX-BGs and agarose (a neutral hydrogel) meant that the release mechanism was primarily diffusion-driven, with concentration gradients driving drug movement.^83^

DOX-BGs-aloe vera initially demonstrated an initial drug release of 21.34% according to Fig. 6(b) until the 6-hour mark. Subsequently, the release rate increased progressively and remained elevated for 48 hours. Between the 6 to 12-hour period, an additional 20.24% of the drug was released, followed by 26.37% between 12 to 24 h, and a further 32.05% between 24 to 48 h; ultimately achieving complete drug release. Among the four hydrogels evaluated, the aloe vera hydrogel demonstrated the highest overall release, reaching 100% cumulative release within the study period. This is problematic because the substantial and rapid drug release observed from DOX-BGs-aloe vera can be attributed to the hydrogel's large pore size, which causes increased surface area, facilitating immediate contact with the external environment and the drug.

Aloe vera hydrogel is mainly composed of neutral polysaccharides such as acemannan, pectin, and glucomannan, which lack ionizable functional groups.^76^ In contrast to polyanionic hydrogels such as sodium alginate, aloe vera does not exhibit charge-induced swelling or electrostatic effects. Instead, its porous nature and high water uptake rate enable rapid diffusion of DOX, resulting in an initial burst release.^76^ Over time, aloe vera hydrogel releases DOX at a constant rate without entering a slow-release stage. Nevertheless, the commercial aloe vera hydrogel employed in this experiment lacked structural stability beyond 24 h and therefore tended to degrade into polymers, thereby increasing its pore size. This structural weakness increased the rate of diffusion of drugs, hence restricting the possibility of obtaining a controlled and sustained drug release.

Fig. 6(b) shows that the sodium alginate hydrogel had an initial cumulative drug release of 12.28% in the first 6 h, as indicated by the pink line. The rate of release was then found to increase in a stepwise fashion, with 16.78% released within 6–12 h, 25.49% within 12–24 h, and 33.76% within 24–48 h. All these increments led to a cumulative drug release of 88.31% in the 48 hours of the study. Ionic exchange, ionization, swelling, and matrix erosion determine the sustained drug-release profile of a sodium alginate hydrogel in pH 7.4 PBS.

In the initial hours (3–6 h), ionic crosslinking between alginate and Ca^2+^ inhibits pore enlargement, thereby slowing drug delivery. As time elapses, Na^+^ ions in the medium around the cross-linked network replace Ca^2+^ ions, weakening the network and making it more porous, thereby allowing drug diffusion. As time elapses, Na^+^ ions in the medium around the cross-linked network replace Ca^2+^ ions, weakening the network and making it more porous, thereby allowing drug diffusion. The hydrogel absorbs solution (PBS), forming a gel layer that regulates diffusion. Instead of acting as a rigid barrier, swelling increases permeability, leading to a gradual increase in drug release. Gradually, the porosity is further widened by matrix erosion, allowing drug diffusion. Carboxyl (–COO^−^) groups are ionized at pH 7.4, and deprotonation causes electrostatic repulsion between negatively charged polymer chains. This increases swelling and promotes further drug release. Beyond 24 h, the polymer network reaches maximum expansion, allowing near-complete drug diffusion as the remaining Ca^2+^ alginate interactions weaken.^77^

### 3.9 Biodegradability and sustainability of the hydrogels

The four hydrogels used in the present study are naturally biodegradable because of their polysaccharide structure. Agar^84^ and agarose^85^ undergo gradual biodegradation. A recent animal study demonstrated that agarose hydrogels at low concentrations break down within 30 days and are replaced by surrounding connective tissue.^86^ Aloe vera hydrogel^76^ has been used in wound healing to support tissue regeneration, as the gel degrades and is replaced by new tissue without long-term persistence.^87^ Sodium alginate hydrogel^88^ has limited long-term stability under physiological conditions, where calcium-cross-linked alginate slowly releases its divalent ions and becomes weaker *in vivo* through a series of exchanges between monovalent and divalent cations, leading to bio-erosion.^89^ Given their limited persistence and lack of tissue accumulation, they are typically safe, short-term biomaterials that can be used for drug delivery.

### 3.10 Compression testing

**Fig. 7:**
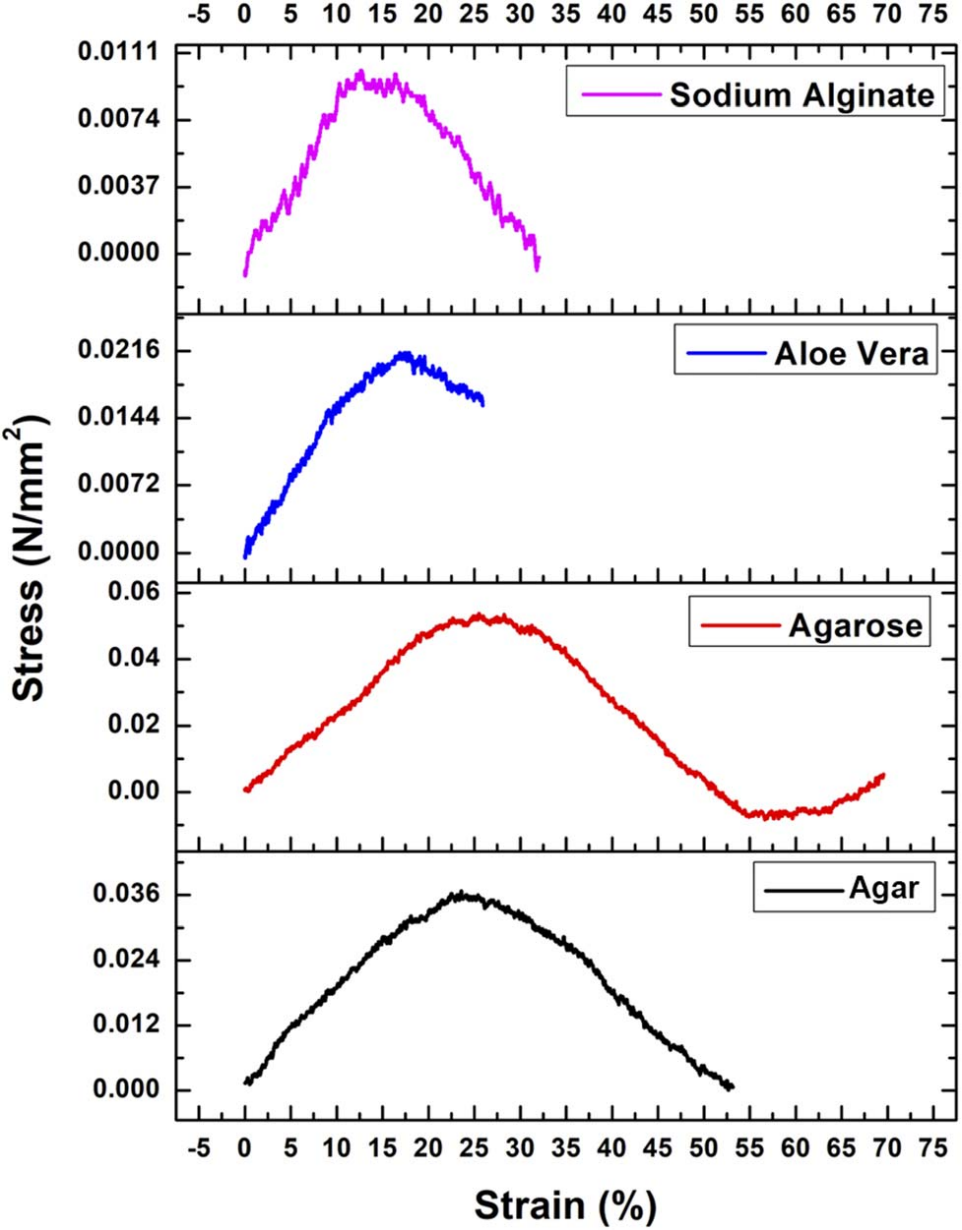
Compressive stress–strain curves of the four hydrogels.

Stress–strain curves generated through compression testing are essential for characterizing the mechanical properties of hydrogels. These curves depict the material's deformation response to applied stress, providing key insights into its elasticity, yield strength, ultimate compressive strength (UCS), and energy absorption capacity. Fig. 7 illustrates the stress–strain curves of the four hydrogels obtained through compression testing.

Agar and agarose hydrogels exhibited similar stress–strain behaviors due to their shared polysaccharide chemical structure. During the compression test, agar hydrogel maintained its structural integrity and exhibited good elasticity, withstanding a high compressive strain of approximately 53% before failure. Agar ranked second strongest in load-bearing capacity, with a UCS of 0.0367 N mm^−2^. Additionally, agar exhibited the second-largest area under the stress–strain curve, indicating its significant energy-absorption capability during compression. In comparison, the yield strength of agarose hydrogel was measured at 0.0276 N mm^−2^.

The agarose hydrogel demonstrated superior mechanical properties, enduring compressive strains of up to 55% before failure. It exhibited the highest ductility and compressive strength amongst all the hydrogels tested, with a compressive strength of 0.0537 N mm^−2^. Moreover, agarose exhibited the largest area under the stress–strain curve, indicating the greatest energy absorption capacity under compression. Its yield strength was measured at 0.0303 N mm^−2^, the highest value observed among the hydrogels analyzed.

Aloe vera hydrogel exhibited a compressive strain of approximately 26% and a UCS of 0.2145 N mm^−2^. Despite its comparatively high UCS, aloe vera displayed the smallest area under the stress–strain curve, reflecting the lowest energy absorption during compression testing. The yield strength, determined using the offset method, was 0.0165 N mm^−2^, lower than that of both agar and agarose hydrogels.

The sodium alginate hydrogel exhibited a UCS of 0.01012 N mm^−2^, significantly lower than that of agar and agarose. The yield strength of sodium alginate was determined to be 0.00945 N mm^−2^, indicating a lower capacity to withstand applied stress before yielding.

All the hydrogels' mechanical behaviour can be explained by the characteristic pressures and solid stresses observed in solid tumors. Interstitial fluid pressure in solid tumors rises up to approximately 20 mmHg,^90^ and solid stresses generated by tumors range between 1–10 kPa,^91^ which are sufficient to squeeze the vessels around them and influence the drug delivery. Thus, the four natural hydrogels agar (36.7 kPa), agarose (53.7 kPa), aloe vera (214.5 kPa), and sodium alginate (10.12 kPa) demonstrated compressive strengths ranging within or above the normal solid-tumor stress range (1–10 kPa) indicating that the hydrogels can withstand mechanical forces associated with tumors and retain their integrity to allow delivery of drugs to the body *in vivo*.

### 3.11 Transport properties of hydrogels

The diffusion behavior of Rhodamine 6G (R6G) dye in hydrogels was examined through a macroscopic approach, leveraging the underlying principle that the diffusion behavior of dyes in a hydrogel can mimic the transport properties of drugs and aid in predicting release profiles before pharmaceutical testing.^92^ The results demonstrated that dye diffusion occurred most slowly in agarose and agar hydrogels, followed by aloe vera and sodium alginate. Fig. S4 presents the results of diffusion experiments, focusing on the diffusion of R6G from the source solution into the gels. These findings were supported by UV-vis spectra,^93^ which showed that agarose hydrogel showed the slowest and sustained dye diffusion over three days. This finding suggests that agarose hydrogel, with its specific pore structure and cross-linked network, was well-suited for efficient drug transport, making it a promising candidate for a drug administration platform. The reported findings align with existing literature on the diffusion properties of molecules in agarose gels, as reported by Monika *et al.*^93^

**Fig. 8:**
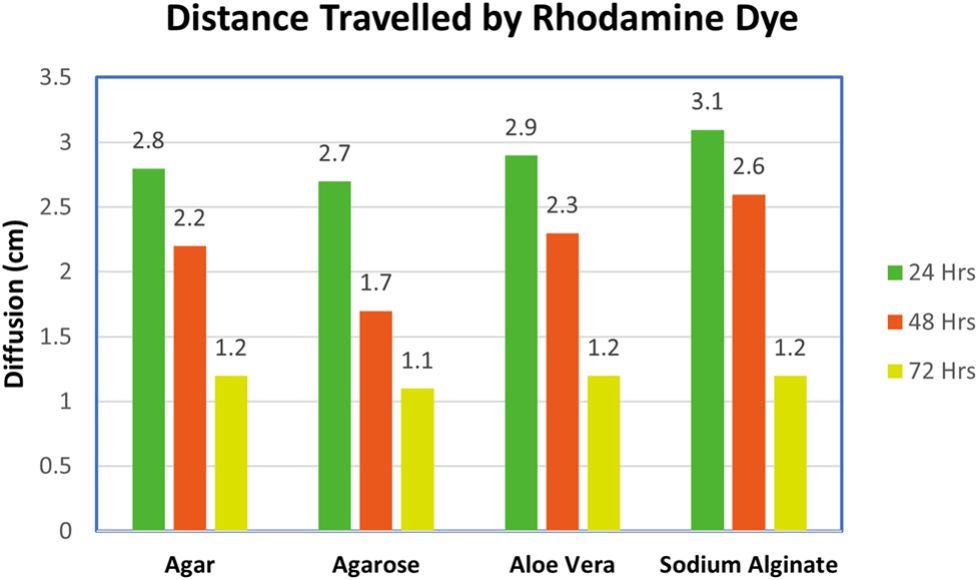
Distance travelled by the Rhodamine-b dye in all four hydrogels recorded in cm.

The transportation dynamics of the hydrogels were assessed by measuring the distances travelled by Rhodamine 6G (R6G) at 24, 48, and 72-hour intervals,^93^ as illustrated in Fig. 8. This data allowed for the calculation of the diffusion rate of R6G dye within the hydrogels. Agarose hydrogel facilitated the least travel distance, making it a noteworthy candidate for effective drug transport within a 72-hour timeframe. A similar conclusion was drawn from the work of Monika *et al.*,^93^ in which pure agarose was found to exhibit stable transport properties, ensuring consistent drug transport.

## 4 Conclusion

This research has provided a comprehensive examination of the potential of BGs as advanced drug delivery vehicles, in combination with various natural hydrogels, specifically agar, agarose, aloe vera, and sodium alginate, for the targeted delivery of the anticancer drug, DOX. The study demonstrated that BGs prepared with Tween-80 exhibited a capsule-like morphology with surface punctures, facilitating a drug encapsulation efficiency of 67%. The DOX-loaded BGs were encapsulated in each of the four hydrogels and evaluated for their chemical composition, mechanical properties, and drug-release profiles. Results revealed the pH-responsive nature of the BGs, with an aggressive burst release of the drug observed at pH 3.0, which was deemed unsuitable for further experimentation. At a more physiologically relevant pH of 6.5, all hydrogels exhibited burst release from 3 to 6 hours, continuous drug release from 6 to 24 hours, and a decline in drug release from 24 to 48 hours. At this pH, among the other three hydrogels, agarose showed the most controlled drug-release profile, resulting in the highest overall drug release of 77.19%. At pH 7.4, agarose showed a slow and nearly constant drug release rate with a cumulative drug release of 38.07%, indicating that the drug does not suddenly release into the bloodstream. This study is limited to *in vitro* analysis, necessitating animal model studies to validate the efficacy of the hybrid system. Further preclinical and clinical investigations are required to confirm its potential for clinical applications. These natural hydrogels do have some limitations, such as lower mechanical strength, variable instability under physiological conditions (temperature, pH), which influence the drug release kinetics of each hydrogel due to the differences in the compressive strength and swelling behavior observed in this study. In addition, the biological source of these hydrogels may vary in composition or molecular weight, which may affect gelation and reproducibility. Another limitation of the study is that the efficiency of chemically induced BG production may vary with bacterial species and chemical agents, potentially affecting yield, structural integrity, and drug encapsulation capacity. The successful integration of BGs with agarose hydrogel, creating the bacterial ghosts-natural hydrogel hybrid system (BG–NH–HS), showcases significant potential for future applications in cancer treatment and beyond. Further research is recommended to investigate the *in vivo* efficacy, including cytotoxicity assays across various cancer cell lines, and to evaluate the efficiency and pharmacokinetics of the BG-HS system. These studies could pave the way for clinical translation and broader therapeutic applications in cancer management and potentially other diseases.

## Conflicts of interest

There are no conflicts of interest to declare.

## Supplementary Material

RA-016-D6RA00738D-s001

## Data Availability

The relevant data supporting this article have been included as part of the supplementary information (SI). Supplementary information: detailed methods and supporting data for bacterial ghost production and validation, doxorubicin loading and encapsulation efficiency (UV-vis calibration and calculations), kinetic modeling (zero-, first-, Higuchi, and Korsmeyer–Peppas models), *in vitro* drug release profiles at different pH values, dye diffusion assays, and associated tables and figures supporting the main manuscript. See DOI: https://doi.org/10.1039/d6ra00738d.
