# Supplementary material for: Bacterial ghosts embedded in natural hydrogels as drug delivery vehicles for cancer treatment
Source: RSC Adv. 2026 Feb 20;16(12):10400–17. doi: 10.1039/d6ra00738d (PMC12922019; doi:10.1039/d6ra00738d)
Supplement: RA-016-D6RA00738D-s001 [file RA-016-D6RA00738D-s001.pdf]

Supplementary Information (SI) for RSC Advances.
This journal is © The Royal Society of Chemistry 2026

SUPPLEMENTARY FILE

Contents

1.0 Characterization and Confirmation of BG Production 2
1.1 Optical Density (OD) Analysis 2
1.2 Viability Testing of BGs 2
2.0 Reference Figure 2
3.0 Extraction of Doxorubicin from Bacterial Ghosts 3
3.1 Encapsulation Efficacy Analysis of the DOX-Loaded-BGs 3
4.0 Kinetic Models of Drug Release 4
4.1 DOX-BGs-Agar 4
4.2 DOX-BGs-Agarose 5
4.3 DOX-BGs-Aloe Vera 5
4.4 DOX-BGs-Sodium Alginate 6
5.0 In Vitro Drug Release 7
6.0 Drug Encapsulation Efficiency 9

1.0 Characterization and Confirmation of BG Production

1.1 Optical Density (OD) Analysis

O.D. measurements indicated that the E. coli culture with an O.D. above 0.4 exhibited poor lysis upon addition of Tween-80. In contrast, E. coli cultures with an O.D. of 0.2-0.3 produced highly effective bacterial ghosts.

1.2 Viability Testing of BGs

The prepared BG pellets were evaluated for viable cells by spreading samples onto Nutrient agar plates. The plates were then incubated at 37 °C in a 5% CO2 incubator for 24 hours. Plates were visually examined for the presence of colonies (Ali et al., 2023).

2.0 Reference Figure

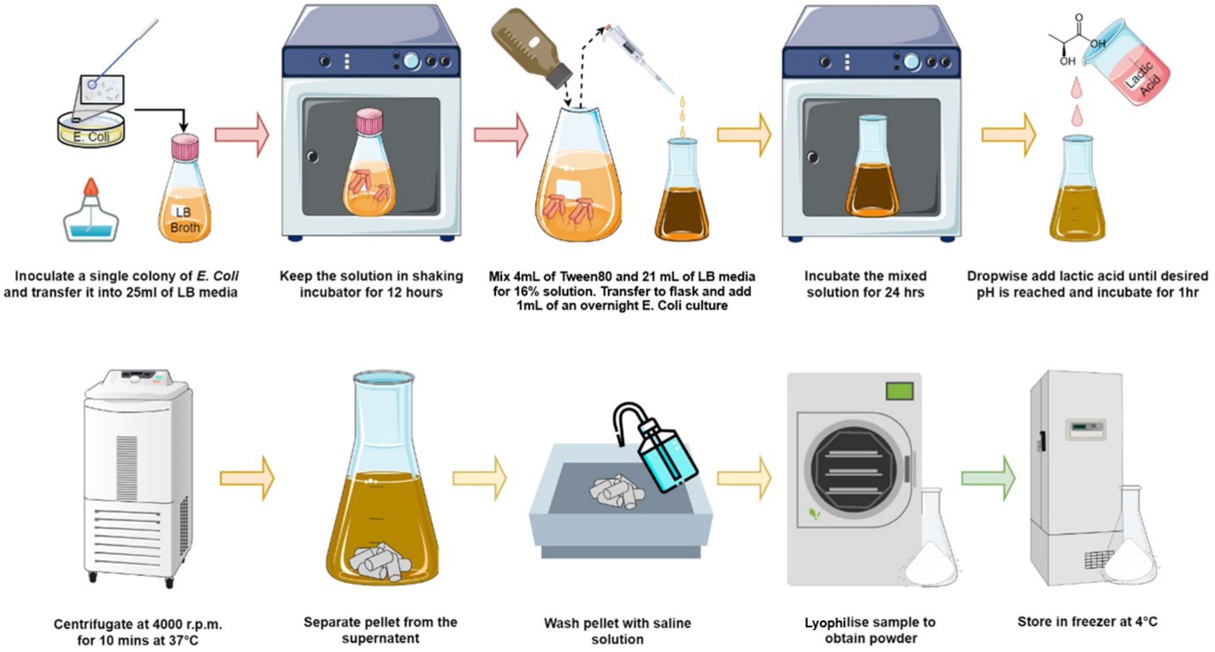

Figure S1: Overview of the Bacterial Ghosts preparation process

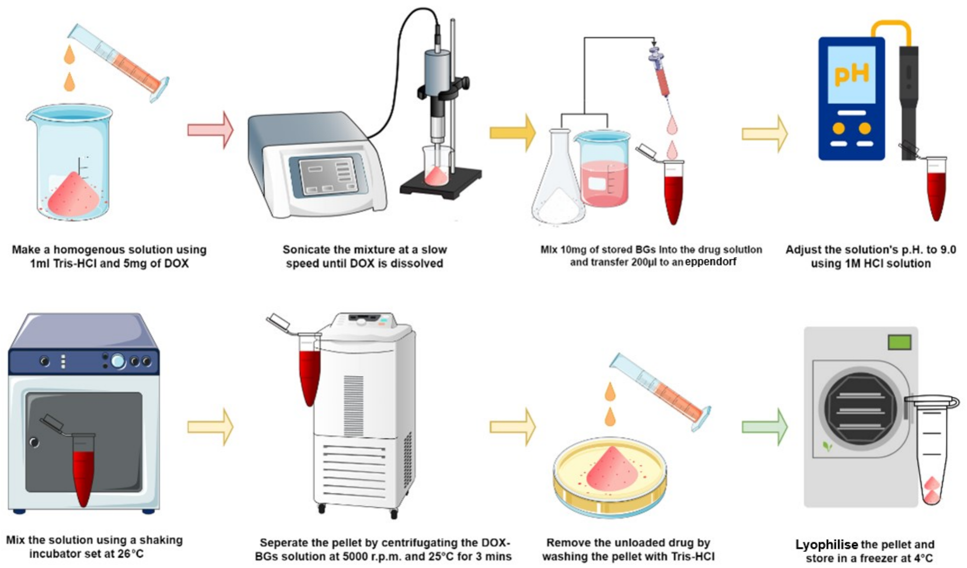

Figure S2: Schematic representation of DOX loading into Bacterial Ghosts

3.0 Extraction of Doxorubicin from Bacterial Ghosts

To determine the amount of DOX present in the BGs, DOX-loaded BGs (10 mg) were first washed with Tris-HCl Buffer five times to remove any unloaded drug. Next, the drug-loaded BGs were suspended in a lysis solution comprising water, sulphuric acid, and sodium dodecyl sulphate. In detail, 10 mg of the washed bacterial cells were first resuspended in 1 mL of distilled water and mixed with 1 mL of 10% SDS solution. The mixture was then incubated at 65°C for 10 minutes. Following this, 1 mL of 10 mM sulfuric acid was added, and the solution was incubated at 65°C for an additional 10 minutes. Finally, the sample was subjected to centrifugation at 4000 rpm for 3 minutes (Paukner et al., 2004), (Rabea et al., 2020), (Sadia, 2021), (Alanazi et al., 2020).

3.1 Encapsulation Efficacy Analysis of the DOX-Loaded-BGs

The DOX concentration in the BGs was determined using UV-Vis Spectrophotometry (Wu et al., 2022) by measuring the extracted supernatant at 470 nm. An indirect approach was used to determine the loading capacity and encapsulation efficiency of DOX within BGs, relying on measuring unbound DOX in the supernatant rather than directly quantifying the drug within the BGs. The following formula was used to calculate the amount of the loaded drug inside BGs.

$$Loading\ Capacity = \frac{W_{DOX}}{W_{BGs}}\ \mu g/mg$$ (Rabea et al., 2020)

DOX calibration curves ranging from 0.2 to 1.0 mg/mL were generated by measuring absorbance at 470 nm using ultraviolet–visible (UV–VIS) spectroscopy. The concentration of DOX in the supernatant was estimated using the calibration curve (Ansary et al., 2025). The reference buffer consisted of the lysis solution prepared as previously described. To ensure accuracy and reproducibility, each measurement was performed in triplicate. The entrapment efficiency (EE%) of DOX encapsulated within the BGs was calculated using the following equation.

$$EE\ \% = \frac{W_S}{W_{Total}} \times 100\ (\frac{\mu g}{\mu g})$$

Where, WS is the weight of loaded DOX in BGs
Where, W_{Total} *is the initial weight of DOX used* (Alanazi et al., 2020).

4.0 Kinetic Models of Drug Release

To determine the drug release mechanism of the formulation, the *in vitro* dissolution data were analysed using various kinetic models (Chandra et al., 2022):

- Zero-Order Model: Evaluates drug release by plotting the cumulative percentage of drug released over time to determine whether the release follows a constant rate, independent of drug concentration.
- First-Order Model: Analyses the log cumulative percentage of drug remaining over time, assessing whether the release rate is proportional to the remaining drug concentration in the matrix.
- Higuchi Model: Examines the relationship between the square root of time and the cumulative percentage of drug released, describing drug release controlled by diffusion from a porous or swellable matrix system.
- Korsmeyer-Peppas Model: Determines the drug release mechanism by plotting log cumulative percentage of drug released against log time, helping to distinguish between diffusion, swelling, and erosion-controlled drug release.

Table S1: Kinetic model of DOX release from BG-HS at pH 6.5

Model	Hydrogels							
	Agar		Agarose		Aloe Vera		Sodium Alginate	
	Slope	R2	Slope	R2	Slope	R2	Slope	R2
**Zero Order**	1.62	0.89	1.61	0.84	1.41	0.93	1.30	0.93
**First Order**	-0.014	0.97	-0.014	0.94	-0.011	0.99	-0.0088	0.98
**Higuchi's**	14.89	0.97	15.03	0.94	12.81	0.99	11.85	0.99
**Korsmeyer Peppas**	1.64	0.82	1.40	0.82	0.95	0.94	1.43	0.91

4.1 DOX-BGs-Agar

The initial burst release is attributed to the high porosity of the Agar hydrogel, which facilitates rapid water absorption and diffusion of surface-associated drug molecules. This behavior aligns with the Higuchi diffusion model ($r^2 = 0.9706$), which describes diffusion-controlled drug release from a porous matrix system. The porous structure of the hydrogel allows for immediate swelling, leading to the rapid initial release of the drug during the early phase. Following the burst phase, drug release progressively slowed as the concentration of the remaining drug decreased, which is consistent with First-Order Kinetics ($r^2 = 0.972$). This controlled release phase, observed between 6 and 24 hours, indicates a concentration-dependent mechanism, where drug diffusion is influenced by its availability within the hydrogel matrix. As more drug is released, the

concentration gradient decreases, thereby reducing the release rate over time. Agar hydrogels are known to exhibit pH-sensitive swelling, influenced by the ionization of carboxylate groups within the polymer network. Although previous studies suggest that swelling is enhanced at higher pH levels, leading to increased drug diffusion, the primary release mechanism observed in this study was diffusion-driven, as confirmed by the strong correlation with the Higuchi model. Additionally, hydrolytic degradation of the hydrogel structure may have contributed to the extended drug release beyond 24 hours, facilitating sustained delivery. The moderate correlation with Zero-Order Kinetics ($r^2 = 0.8925$) suggests that while some level of sustained release was observed, the release rate was not constant over time, ruling out Zero-Order as the dominant mechanism. Furthermore, the Korsmeyer-Peppas model ($r^2 = 0.8178$) did not provide the best fit, indicating that swelling and polymer relaxation played a minimal role in drug release. Instead, diffusion through the hydrogel matrix remained the primary release mechanism.

4.2 DOX-BGs-Agarose

The progressive decrease in the drug release rate over time aligns with First-Order Kinetics ($r^2 = 0.9392$), which suggests that the release rate is dependent on the remaining drug concentration within the hydrogel matrix. As the available drug within the hydrogel depletes, the concentration gradient driving diffusion decreases, resulting in a gradual reduction in the release rate. This pattern confirms that drug release from Agarose hydrogel is not uniform but slows down as the drug reservoir is exhausted, characteristic of first-order kinetics. Furthermore, the Higuchi model ($r^2 = 0.943$) provided the best correlation, indicating that drug release is primarily governed by diffusion through the porous hydrogel matrix. Since Agarose does not have strong electrostatic interactions with DOX, and its swelling behavior remains largely pH-independent, drug release is not significantly influenced by polymer relaxation or degradation but rather by Fickian diffusion. The consistent decline in release rate over time supports a diffusion-controlled mechanism, where the drug is gradually released as it migrates from the hydrogel matrix into the surrounding medium. The moderate correlation with Zero-Order Kinetics ($r^2 = 0.8432$) suggests that while Agarose hydrogel does provide some level of sustained release, the overall release pattern does not follow a constant rate over time, ruling out zero-order as the dominant mechanism. Additionally, the weaker correlation with the Korsmeyer-Peppas model ($r^2 = 0.8212$) confirms that swelling and polymer relaxation effects played a minimal role in drug release, further reinforcing that diffusion through the hydrogel was the primary release mechanism.

4.3 DOX-BGs-Aloe Vera

The Higuchi model ($r^2 = 0.9922$) provided the best correlation, confirming that drug release from the Aloe Vera hydrogel was primarily governed by diffusion through the porous matrix. The neutral polysaccharide composition of Aloe Vera, including acemannan and glucomannan, lacks significant protonation or deprotonation sites, leading to minimal pH-responsive swelling behavior. This ensures that drug release is largely independent of pH fluctuations, with diffusion acting as the dominant release mechanism rather than swelling or polymer relaxation. Additionally, as the hydrogel matrix gradually degraded, the drug continued to diffuse, though at

a slower rate, due to the weakened structure and reduced hydration capacity.The strong correlation with First-Order Kinetics (r^2 = 0.9913) further supports that drug release was concentration-dependent, meaning that as the drug reservoir within the hydrogel depleted, the release rate also decreased. This aligns with the observed transition from an initial burst phase to a more controlled and steady release phase, followed by a final phase of slow drug release. The gradual reduction in drug concentration available for diffusion over time further reinforces first-order behavior, where the rate of release is directly proportional to the remaining drug concentration in the hydrogel matrix. Although the Zero-Order model (r^2 = 0.9349) showed some correlation, it did not fully explain the release mechanism since drug release was not constant over time. Instead, a declining release rate was observed, ruling out a purely zero-order process. Furthermore, the Korsmeyer-Peppas model (r^2 = 0.943) exhibited a weaker correlation, indicating that swelling and polymer relaxation had minimal impact on drug release. This confirms that the release process was not significantly influenced by matrix swelling but was predominantly governed by diffusion through the hydrogel network.

4.4 DOX-BGs-Sodium Alginate

The Higuchi model (r^2 = 0.9887) provided the best correlation, indicating that drug release from Sodium Alginate hydrogel was primarily governed by diffusion through the porous matrix. The pH-dependent swelling behavior of Sodium Alginate, driven by the ionization of carboxyl ($-COO^-$) groups, played a critical role in regulating drug release. At pH 6.5, which is slightly above the pKa (~3.5–5.5) of alginate, moderate ionization occurred, leading to electrostatic repulsion and controlled swelling. This prevented hydrogel shrinkage and maintained a consistent pore size, ensuring gradual but sustained drug release beyond the burst phase. Additionally, ionic crosslinking between Sodium Alginate and divalent cations (Ca^{2+}) stabilized the hydrogel structure, further restricting drug movement and prolonging the release phase.The strong correlation with First-Order Kinetics (r^2 = 0.9789) further supports that drug release was concentration-dependent, meaning that as the drug reservoir within the hydrogel depleted, the release rate also decreased. This aligns with the observed transition from an initial slow burst phase to a more controlled and steady release phase, followed by a final phase of slow drug release. The gradual reduction in drug concentration available for diffusion over time further reinforces first-order behavior, where the rate of release is directly proportional to the remaining drug concentration in the hydrogel matrix. Although the Zero-Order model (r^2 = 0.9331) showed some correlation, it did not fully explain the release mechanism since drug release was not constant over time. Instead, a declining release rate was observed, ruling out a purely zero-order process. Furthermore, the Korsmeyer-Peppas model (r^2 = 0.9054) exhibited a weaker correlation, indicating that swelling and polymer relaxation had minimal impact on drug release. This confirms that the release process was not significantly influenced by matrix swelling but was predominantly governed by diffusion through the hydrogel network.

5.0 In Vitro Drug Release

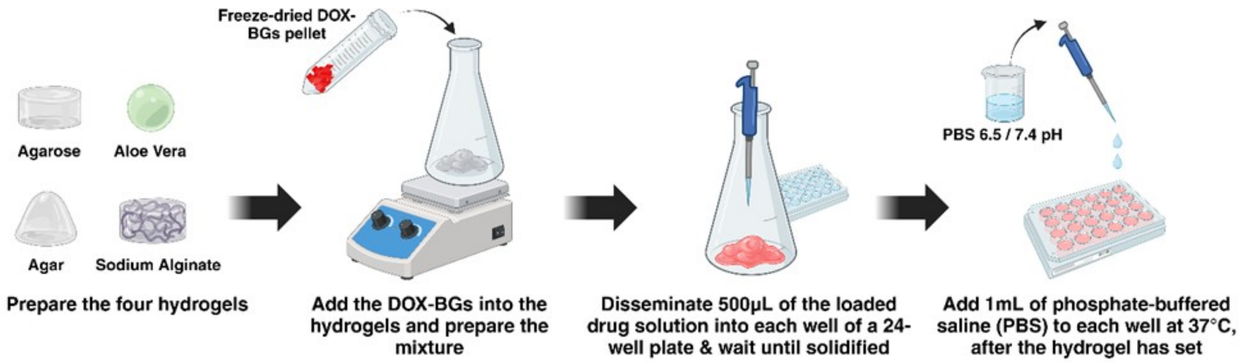

Figure S3: In Vitro drug release profiles from BG-HS at pH 6.5 and pH 7.4

Table S2: In vitro drug release from Bacterial Ghosts

pH	Time (hr)	Absorbance	Percentage Drug Release	Cumulative Drug Release
**3.0**	3	0.25	7.26	7.26
	6	0.59	19.11	26.37
	12	0.62	20.14	46.51
	24	0.63	20.63	67.14
	48	0.63	26.59	93.73
**6.5**	3	0.22	6.41	6.41
	6	0.42	13.21	19.61
	12	0.49	15.61	35.22
	24	0.60	19.39	54.61
	48	0.71	23.45	78.06
**7.4**	3	0.22	6.13	6.13
	6	0.35	10.88	17.01
	12	0.42	13.43	30.44
	24	0.55	17.85	48.30
	48	0.61	19.89	68.19

Table S3: Cumulative drug release of DOX from hydrogels at pH 6.5

Hydrogel	Time (hr)	Absorbance	Percentage Drug Release	Cumulative Drug Release
**Agar**	3	0.056	0.57	0.57
	6	0.37	11.52	12.09
	12	0.60	19.57	31.66
	24	0.82	27.17	58.83
	48	0.52	16.57	75.41
**Agarose**	3	0.075	1.23	1.23
	6	0.46	14.59	15.82
	12	0.67	21.80	37.62

	24	0.83	27.62	65.25
	48	0.38	11.94	77.19
**Aloe Vera**	3	0.18	4.76	4.76
	6	0.35	10.75	15.51
	12	0.48	15.18	30.69
	24	0.60	19.64	50.33
	48	0.63	20.58	70.91
**Sodium Alginate**	3	0.017	-0.79	-0.79
	6	0.27	8.17	7.38
	12	0.45	14.34	21.73
	24	0.63	20.58	42.31
	48	0.54	17.34	59.65

Table S4: Cumulative drug release of DOX from hydrogels at pH 7.4

Hydrogel	Time (hr)	Absorbance	Percentage Drug Release	Cumulative Drug Release
**Agar**	3	0.076	1.26	1.26
	6	0.15	3.88	5.13
	12	0.38	12.02	17.15
	24	0.61	20.00	37.15
	48	0.85	28.20	65.34
**Agarose**	3	0.095	1.93	1.93
	6	0.19	5.14	7.07
	12	0.27	7.96	15.03
	24	0.34	10.51	25.54
	48	0.40	12.53	38.07
**Aloe Vera**	3	0.27	7.96	7.96
	6	0.42	13.39	21.34
	12	0.62	20.24	41.58
	24	0.80	26.37	67.95
	48	0.97	32.56	100.51
**Sodium Alginate**	3	0.16	4.04	4.04
	6	0.28	8.24	12.28
	12	0.52	16.78	29.06
	24	0.77	25.49	54.55
	48	1.01	33.76	88.31

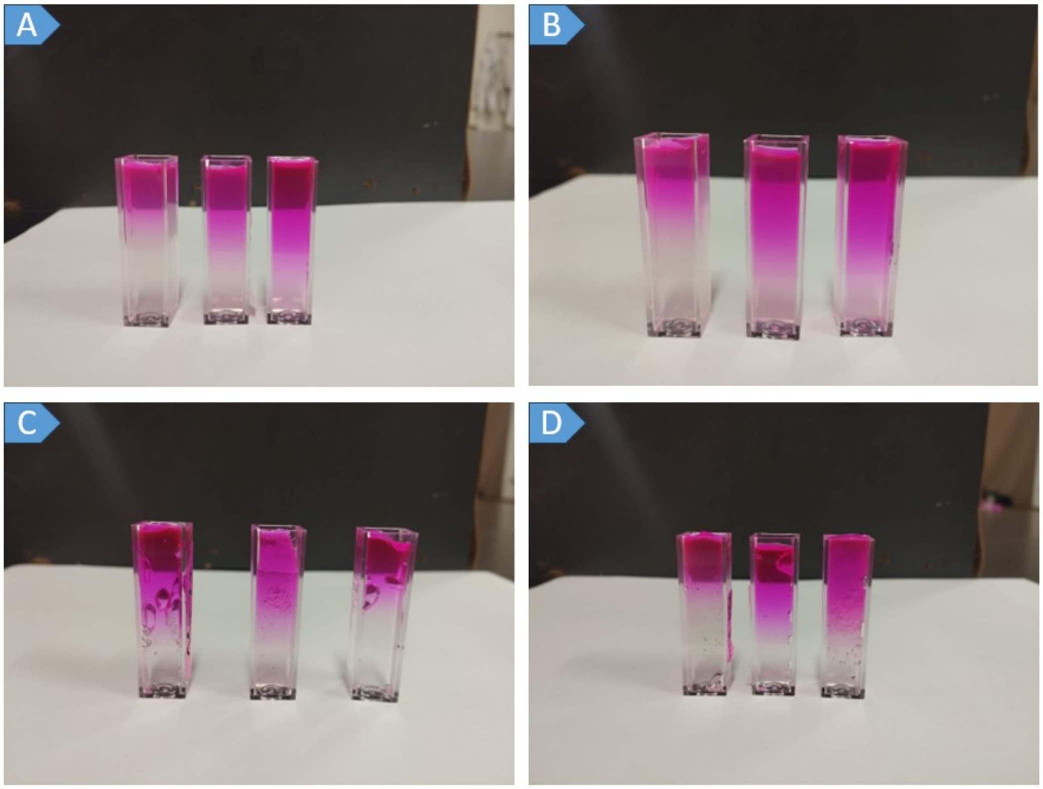

Figure S4: Dye diffusion assay of (a): Agar, (b): Agarose, (c): Aloe Vera, (d): Sodium Alginate at 24, 48, & 72 hrs.

6.0 Drug Encapsulation Efficiency

Using a UV-VIS spectrophotometer, the absorbance was measured at 470nm for different concentrations of Doxorubicin. The resulting standard curve is shown in Figure S1. The initial concentration of Doxorubicin used was 1 mg/ml. Equation S1 was used to determine the drug concentration loaded into the BGs.

$y = mx + b$

Thus, $\frac{y-b}{m} = x$

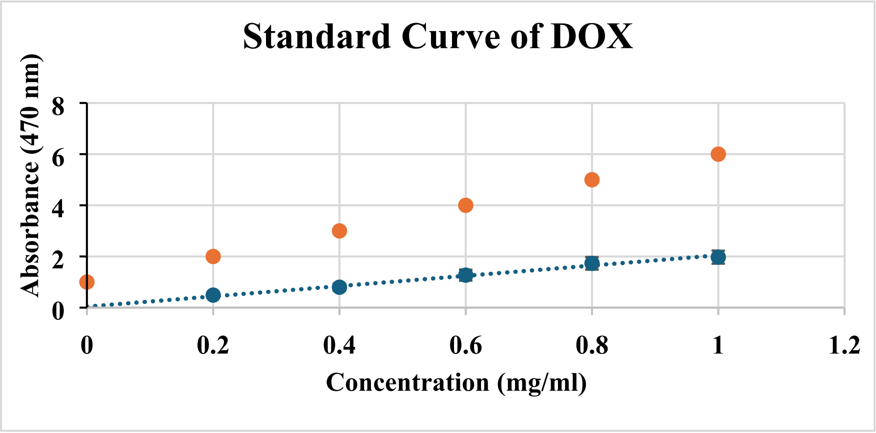

Figure S5: Standard Curve of Doxorubicin

Table S5: Absorbance recorded at 470nm for the released DOX in the medium

OD-1	OD-2	OD-3	Average	SD	SK in medium
1.39	1.41	1.39	1.40	0.0094	0.678

$$y = mx + b$$

$$1.395 = 2.0079x + 0.0396$$

$$x = 0.675\ mg/ml$$

Table S1 presents the absorbance values obtained from three distinct runs of BGs. To improve precision, the average of the Y values (absorbance) is used, where Y denotes the absorbance obtained via UV-Vis Spectroscopy. Only BGs that released 1400 ng/µl of DNA were selected. The selected pellets were then broken, and their UV-absorbance values were measured. The concentration of the drug released into the medium is calculated from the DOX standard curve, which is subsequently used to determine the encapsulation efficiency.

$$Encapsulation\ Efficiency\ (\%) = \frac{Drug\ Released\ in\ the\ medium}{Concentration\ of\ Doxorubicin\ added\ initally} \times 100$$

$$= \frac{0.675}{1} \times 100 = 67.5\% \quad \text{.... (S1)}$$

Therefore, the Encapsulation Efficiency (%) = 67.5%
